# A qualitative study of the impact of the World Health Organisation QualityRights human rights and mental health Training on changing attitudes to mental health and human rights in Ghana

**DOI:** 10.3389/frhs.2026.1673311

**Published:** 2026-02-05

**Authors:** Thea Sobers, Leveana Gyimah, Sally-Ann Ohene, Martin Orrell, Emma Poynton-Smith, Ling Wang, Joana Ansong, Florence Baingana, Maria Francesca Moro, Sarah Sackey, Emmanuel Fokuo, Pinaman Appau, Nathalie Drew, Michelle Funk

**Affiliations:** 1 Institute of Mental Health, University of Nottingham, Nottingham, United Kingdom; 2 WHO Country Office, Accra, Ghana; 3 WHO Regional Office for Africa, Brazzaville, Democratic Republic of Congo; 4 Columbia University Mailman School of Public Health, New York, NY, United States; 5 Christian Health Association of Ghana, Accra, Ghana; 6 Ghana Health Service, Accra, Ghana; 7 Ghana Ministry of Health, Accra, Ghana; 8 Policy, Law and Human Rights, Department of Mental Health and Substance Abuse, World Health Organization, Geneva, Switzerland

**Keywords:** attitudes, human rights, mental health, services, WHO, Ghana

## Abstract

Negative and stigmatising attitudes towards human rights and mental health exist worldwide. The Ghanaian government has attempted to tackle such discriminatory attitudes and practices to align with a human rights-based approach in mental healthcare through the implementation of the World Health Organisation QualityRights Initiative in Ghana. As part of the initiative, the World Health Organisation has developed a range of capacity building tools on mental health, disability, human rights and recovery. National stakeholders in Ghana completed the WHO QualityRights e-training to build capacity and change attitudes to promote recovery and respect for human rights for people with mental health conditions, psychosocial and intellectual disabilities. Participants completed pre- and post-training questionnaires assessing perceived attitude and practice change. Thematic analysis was conducted on the open-ended questions on the post e-training questionnaires to discover how attitudes were impacted by the e-training. Three themes emerged: “legal capacity and the right to decide”, “coercion, violence and abuse” and “equality and community inclusion.” Attitudes on the right to make decisions, treatment of individuals with mental health conditions, psychosocial and intellectual disabilities, equality, social inclusion and non-discrimination had improved to be more in line with a human rights-based and recovery-oriented approach to mental health. The QualityRights training shows promise as a scalable intervention to reduce stigma, promote rights, and improve care for individuals with mental health conditions, psychosocial and intellectual disabilities.

## Introduction

People with lived experience of mental health conditions and psychosocial and intellectual disabilities experience violations to their human rights across the globe. In many countries, those with lived experience face restrictions of their civil, cultural, economic, political and social rights, lack of access to mental health services, abuse and coercion in residential facilities and places of detention and restrictions to the exercise of legal capacity (1–3). Coercive practices can include the use of restraint, seclusion, involuntary treatment, admission or detention, substitute decision making or involuntary contraception, sterilisation or abortion amongst others (4–8). These practices can occur within or outside of mental health settings and are all in conflict with human rights-based mental health care (4). Moreover, coercive practices and abuses in human rights can be legitimised, approved and extensively used as part of mental healthcare in many countries in both hospital and community settings. The human rights violations that many people experience are in part a consequence or reflection of the attitudes shaped by various factors including culture, media representation, mental health literacy, training, and education (9–11). Among many stakeholders, there is substantial support for several human rights restrictions (12). Furthermore, mental health professionals (MHPs), a key stakeholder group, often hold attitudes that view coercive practices as beneficial and necessary and can harbour strong stereotypes about mental health and a desire for greater separation from those with lived experience of mental health conditions (13–15). In Ghana, high rates of coercive practices have been found within mental healthcare: chemical and manual restraint, seclusion and forced admission and treatment have been reported (16–19). When using alternative services like prayer camps, human rights violations like forced admission and treatment, inhumane conditions, chaining, denial of food, detention and verbal abuse have also all been reported (20, 21) leaving little to no choice to use non-coercive services.

In Ghana and many other countries, mental health conditions are often viewed in the context of spiritualism, leading to attitudes that are formed around traditional beliefs like being cursed or invaded by evil spirits (16, 22–24). People with mental health conditions can be perceived as violent, dangerous, unpredictable or to be denied their basic rights (23, 25). A recent study in Ghana found that attitudes towards people with mental health conditions, psychosocial and intellectual disabilities did not align with a human rights-based approach to mental health. Support for the use of coercive practices was common and overall, MHPs and family members were seen as being in the best position to make treatment decisions on patient's behalf. This belief was upheld by health and MHPs, although less so than other stakeholder groups (26). These stigmatising attitudes can lead to human rights violations and discriminatory practices like social exclusion, maltreatment, shame and problems with family and personal relationships (16, 23, 27), reduced recovery and delayed help seeking (28).

The United Nations Conventions of Rights of Persons with Disabilities (CRPD) (29) presents international human rights standards that need to be upheld for persons with disabilities. It presents a solid foundation for countries to develop and implement policies and campaigns that address the effects of stigma and discrimination. The CRPD has been ratified in several countries (30) and these countries have obligations to promote and protect the human rights of all persons with disabilities including those with mental health conditions and psychosocial and intellectual disabilities (29). The CRPD framework is central to the World Health Organisation (WHO) QualityRights Initiative, which is focused on recovery and human rights. The initiative proposes practical solutions to improve the standard of care and practices within mental health services and provide and promote respect for the human rights of individuals with lived experience of mental, psychosocial, intellectual and cognitive disabilities (30, 31) with a focus on freedom from coercive practices, respect for legal capacity and promotion of autonomy, choice, community inclusion and recovery, thereby supporting countries to actualise the rights of the CRPD (32). It uses different strategies like capacity building, service assessment, civil society engagement and policy reform (30, 33). To build capacity and promote human rights, WHO published training and guidance materials covering topics related to mental health, disability, human rights and recovery (34). Attitude changes have been found following the implementation of the QualityRights face-to-face training in India where attitudes towards persons with lived experience improved (35) and in Iceland where there were positive attitudinal changes around issues related to legal capacity, the use of coercion, independent living in the community and treatment (36). Moreover, an evaluation of the e-training found attitudinal shifts towards a human rights-based approach and legal capacity (37).

The Ghanaian government has set out to make improvements within the mental healthcare system and have ratified the CRPD (38) and introduced the Mental Health Act, which emphasised the provision of quality mental health care and action against stigma and discriminatory attitudes in mental health (39). However, challenges have occurred with its implementation (9) and the Act still permits the use of coercive practices like substitute decision making, seclusion, restraint and involuntary treatment (40). This led to the national implementation of the WHO QualityRights Initiative (33) to align mental health and social care with the CRPD. The WHO Assessment Toolkit has been used to assess several psychiatric facilities in Ghana (17, 41), and the QualityRights e-training has also been rolled out country wide to national stakeholders, who have successfully completed the e-training, received their WHO certificate and some have become recognised as e-training coaches (33, 42). By 2022, 22,091 people across the world had completed the QualityRights e-training and received their certifications (26). This included 5,161 people from Ghana where 4,285 participants completed the baseline questionnaires, and 876 completed the follow up questionnaire measures (43) but not all of these provided text comments.

Previous evaluations of WHO QualityRights training in Ghana included a quantitative analysis of attitudes before and after training (26, 43). Overall here was a 40% improvement in attitudes to human rights between baseline and follow up which was highly significant changes In particular, attitudes improved in terms of treatment choice, legal capacity, and coercion. This change was not affected by age, gender, or background experience. This indicated the QualityRights e-training programme was effective in changing people's (especially healthcare professionals') attitudes towards people with mental health conditions and psychosocial, intellectual, or cognitive disabilities. The aim of the current study was to explore qualitative changes in attitudes for people who took part in the QualityRights e-training.

## Methods

The current study builds upon previous evaluations of the QualityRights Initiative in Ghana. This study explored the impact of the e-training on attitudes towards people with a mental health condition, psychosocial and intellectual disability as rights holders with qualitative text data on attitude change being the focus of analysis.

### Design

A qualitative design was employed, using thematic analysis to identify themes. In accordance with the study's aims, qualitative analysis was the most appropriate choice for gaining a deeper understanding of the aspects of attitudinal change elicited by the e-training.

### Participants

The QualityRights Initiative in Ghana was implemented by key stakeholder groups within the Ghanaian mental health sector. These groups worked alongside each other to promote and disseminate the e-training among their networks in Ghana and foster participation in the training. These groups included the Mental Health Authority Ghana, Mental Health Society of Ghana, Ghana Health Service, MindFreedom Ghana, BasicNeeds Ghana, Ta-Excel Foundation, Inclusion Ghana, Special Olympics, Passion for Total Care, the Christian Health Association of Ghana and WHO Ghana. This endeavour led to a mixed profile of participants including people with lived experience, individuals from non-governmental organisations (NGOs), disabled persons organisations (DPOs) and faith-based organisations, health and MHPs, ministry of health representatives, academics including teachers and students and policy makers. All participants for this study completed the WHO QualityRights e-training, voluntarily completed the post-training questionnaire and consented to the use of their data for research evaluation.

One thousand and eighty-two individuals completed the follow-up questionnaire, and more than half of these individuals (*n* = 716) provided text answers to open-ended questions. Their ages ranged from 16 to 69 years old. Eligibility criteria were not required for the current study and all those who completed and consented in the post-training questionnaire were included. Individuals were excluded if consent for their data to be used for research purposes was not obtained.

### Ethical approval

This WHO study received ethical approval from the University Hospital of Cagliari in May 2019 and approval from the Ghana Health Service Ethical Review Board in October 2019. Participants were informed of the aims of the training and its evaluation. Assurance was given concerning confidentiality and anonymity and information regarding voluntary participation, early withdrawal and cessation of participation was disseminated. The fact that the questionnaire was delivered online and anonymously will hopefully have reduced the potential conformity or social desirability bias. Contact details of a key organiser of the QualityRights initiative were also provided. Furthermore, data collection and processing were carried out in line with articles 6 and 9 of the European Regulation 679, which was also translated to participants. Consent was obtained using the online consent form. Then, participants were again reassured of confidentiality and anonymity before completing the post-training questionnaire. An online platform created by the WHO for data collection was used for the consent form, e-training and post-training questionnaire.

### Materials

Data were collected using a post-training questionnaire—developed for specific use following completion of the QualityRights e-training programme. The questionnaire contained a section related to demographics, Likert scale measures of how strongly participants agreed with statements relating to attitudes about the human rights of individuals with mental and psychosocial disabilities and other items related to the content, delivery and quality of the training. Additionally, 7 open ended questions were designed to elicit answers related to perceived attitudinal change, future practice change, e-training effects and feedback about the course platform, modules and quality. The current study utilised written answers to the question “If your attitude towards people with psychosocial, intellectual and cognitive disabilities has changed, please describe in what ways. If your attitude has not changed, please describe why not”. The post questionnaire data used for this study were collected between February 2019 and October 2021.

### Procedure

E-training comprised of six modules that cover mental health, recovery and community inclusion. These were “Human rights”, “Human rights, mental health and disability”, “Legal capacity and the right to decide”, “Ending coercion, violence and abuse”, “Quality services and community inclusion” and “Mental health, well-being and recovery”. In the first two modules, the focus was on human rights in general and in relation to mental health and disability while the other modules focused on the promotion of these rights in mental health and related services. QualityRights e-training can be accessed using the following link: https://humanrights-etrain-qualityrights.coorpacademy.com/signup. The modules took between 8 and 24 h to complete, depending on the prior knowledge of the person completing the training, and the extent to which they completed the additional in-depth materials of the course. Following completion of the e-training, participants were asked to fill out a post-training questionnaire.

### Analysis

Qualitative data was analysed using six-step thematic analysis (43) familiarisation, generating initial codes, searching for themes, reviewing themes, defining and naming themes and producing a final report. Familiarisation with the data occurred through reading the post-questionnaire statements related to attitudinal change and making initial notes from the data. Following this, codes were generated for the statements with a coding framework that was drawn from the data, leading to codes such as “equal rights” and “not a threat”. These codes were reviewed a total of four times until no more codes could be generated from the data. Once coding was complete, codes were mapped and clustered together to search for and identify themes and sub-themes. Each sub-theme was summarised with representative quotes and frequencies. After this, themes were reviewed and refined leading to the creation of subthemes and then refined, generating the overarching themes which represented important ideas that were emphasised across the data and portrayed the aims of the research. These themes were organised deductively within the broad frame of reference of the CRPD, and the final set of themes were reached by consensus (agreement with Author 4, Author 5, and Author 6) through a deductive process based on content analysis and the existing framework of CRPD human rights. These stages allowed data to be reviewed, triangulated, and discussed through different lenses, thus enhancing the credibility of the results, improved reliability and reduced potential bias.

## Results

Of the 716 individuals who provided open-ended responses, 376 (52.5%) quotes related to attitudinal change. Participants' ages ranged between 16 and 61 years old with 48% female. Over half of the sample were affiliated with the Ministry of Health including MHPs, while 82 (21.8%) individuals were service providers and 39 (10.4%) were affiliated with academia. Twenty-seven (7.2%) were from Non-Governmental Organisations, 5 (1.3%) from WHO, 3 (0.8%) from professional associations and 1 (0.3%) from Disabled People's Organisations. Other groups including the Mental Health Authority, Ghana Health Service, Christian Health Association and Ghana Institute of Management and Public Administration represented 16 (4.2%) individuals from the sample.

### Themes emerging from the post e-training questionnaire

Quotes related to attitudes, which were categorised initially according to the CRPD themes: (1) legal capacity and the right to decide; (2) coercion, violence and abuse; (3) equality and community inclusion (Table 1). Analysis revealed a shift in attitudes that aligned with a human rights based approach and were in line with several articles of the CRPD. Many participants expressed attitude changes related to human rights, decision making, coercive practices and treatment, equality, stigma and non-discrimination.

**Table 1:** Themes and subthemes related to attitude change that emerged from the data.

Themes	Subthemes
Legal capacity and the right to decide	Legal capacity
	Autonomy
	Informed consent
	Supported decision making
Coercion, violence and abuse	Coercive practices
	Poor treatment
	Improved treatment
Equality and community inclusion	Equality
	Competency
	Recovery
	Empathy and understanding
	Risk
	Inclusion in society

### Legal capacity and the right to decide

One hundred seven quotes (28%) noted that participants' attitudes had changed in relation to people with mental health conditions and psychosocial disabilities and their right to make decisions about their life, treatment and care.

“I now understand they also have the right to decide on their life”—Mental health or related practitioner

Several comments concerned the ways that people can exercise their right to make decisions, focusing on informed consent and supported decision making. Many participants stated that individuals with lived experience should be included in the decision making and have an active and participatory role in making or influencing decisions about their life, treatment and recovery and should be given the chance to do so.

“… they have a voice and need to be heard especially in their choice of treatment and what’s best for them.”—Policy Maker/Analyst

“People with psychosocial, intellectual and cognitive disabilities should… be given the chance to take part in decisions regarding their treatments…”—Health practitioner

Additionally, participants now also favoured autonomy, stating that the training helped them to understand that service users should be leading their treatment and recovery. Participants stated that they learnt about service user's rights to accept or refuse treatment.

“…people with disabilities should have full agency over all decisions…”—Mental health or related practitioner

“Personally I’ve always believed people with mental disabilities shouldn’t be the final decision makers with regards to their health and personal lives but that perception of mine has changed after this course”—Mental health or related practitioner

Participants also felt differently about the role of mental health professionals, families and carers in the decision-making process, and felt that these people don't always know best. Subsequent to the course they favoured supported decision making, where families and practitioners simply support persons with mental health conditions and psychosocial and intellectual disabilities to make decisions, thereby allowing their right to decide. This was described as better because it permitted persons with lived experience to use their voice and assert their needs.

“… initially I believed that psychiatrists should choose what treatment they feel is best for the patient. Now I know that the steps should be supported not substituted”—Health practitioner

“…the ultimate and best way to help a patient is to assist him or her to make the best choice in his or her recovery.”—Mental health or related practitioner

### Coercion, violence and abuse

Seventy quotes (19%) focused on coercion, violence and abuse. Participants felt that individuals should not be treated poorly or subjected to abuse because of their lived experience.

“… I used to think that when you are mentally challenged, you have no right and can be mishandled but I got to realize that being mentally, psychosocially, and cognitively challenged does not mean you can be mishandled or maltreated…”—Mental health or related practitioner

Participants now opposed the use of coercive practices, even in times of crisis and considered methods such as seclusion, isolation, restraint, force and involuntary treatment and detainment to be wrong, harmful, not a form of treatment, abusive and a violation of human rights. Additionally, a few participants expressed concern about national laws which they now felt endorsed coercion and allowed for the violation of human rights.

“At first I thought seclusion is a best practice, but now I realise it’s a form of rights abuse.”—Mental health or related practitioner

“I now think it is unacceptable to control, coerce or force service users into anything against their will”—Academic

“Now I do understand that our national laws that support involuntary commitment to service is coercive…”—Mental health or related practitioner

Participants now felt people with lived experience should not be treated violently or abused and overall, suggested that they should be treated with respect, dignity and care. The importance of supportive measures was also highlighted.

“…they need to be treated with respect and dignity…”—Academic

“It has changed in a way that, I understood that it is not only medication that can help but support”—Family member or care partner

### Equality, participation and community inclusion

In total, 335 quotes (89%) addressed equality and community inclusion with shifts in attitudes towards fair treatment and equal opportunity. Many participants stated that they now perceived individuals with lived experience as human beings with worth and not just “sick” individuals. Other comments suggested attitude change in relation to common stereotypes about persons with mental health conditions, psychosocial and intellectual abilities. Participants' views surrounding competency changed whereby they now felt that those with lived experience have equal abilities and could better understand their situation and make decisions, perform tasks and live independently. Many participants no longer felt that they were dangerous, violent or a threat. Attitudes shifted about the risk of mental health conditions, where participants felt that anyone could experience a mental health condition.

“I used to pity them and think that they need alms to survive, they cannot do much due to their disabilities. Now I know they can equally achieve greater things as any other people if they are giving opportunities”—Person with other disabilities

“…they are human and entitled to human rights just like any other normal person. Having a mental health condition does not render a person less important, or dangerous to the society.”—Mental health or related practitioner

Moreover, attitudinal shifts related to recovery were also expressed where recovery was seen as possible for persons with lived experience of mental health and psychosocial disabilities and its efficacy was dependent on a number of factors.

“…such disabilities can be reversed with time and through the adaptation of correct practices”—Academic

Equality and non-discrimination were highlighted throughout several comments about human rights. Prior to the e-training, some participants didn't believe certain rights applied to people with mental health conditions and psychosocial and intellectual disabilities but now considered human rights to be applicable to all individuals regardless of mental health status. Attitudes now favoured the respect, protection and fulfilment of their rights, opinions and decisions on an equal basis with others, especially due to their higher susceptibility of abuse.

“I now see reasons why we're all the same and we are all equal, equal rights, privileges and choices in life no matter who or how we are. We are to be treated equally.”—Mental health or related practitioner

Differences were acknowledged, recognised and respected, as participants reported more positive, empathetic and understanding attitudes towards these individuals.

“Now I understand and know where they are coming from.”—Academic

“I have developed a better understanding and feelings of empathy, for people with psychosocial, intellectual and cognitive disabilities”—Person with lived experience

There were attitude changes around stigma and discrimination with participants now seeing stigma as wrong and being opposed to discriminatory practices. They no longer felt the need to avoid or distance themselves from persons with mental health conditions and psychosocial disabilities. They recognised individuals with lived experience as part of society and were in favour of their inclusion and chance to lead normal lives by contributing to development, working and participating in social activities.

“Initially I used to be afraid of people with psychosocial, intellectual and cognitive disabilities and I did not want them close to me but after this program I have come to realise that they were normal just like us and they are not scary as we perceived them to be and they can also live in the society without causing harm to anyone.”—Academic

Additionally, participants expressed ideas about personal responsibility and duty to support equality and community inclusion through advocating and fighting for the rights of individuals with lived experience, assisting with their recovery and supporting their inclusion in society.

“…the need for advocacy and fight for change in our current systems is now and it can begin with me”—Student

### No perceived attitudinal change

For a small number of participants, there were no shifts in attitudes mostly because people felt they were already following good practice in line with the QR training and were knowledgeable about human rights.

“My attitude has not really changed because I have been practicing most of these in my care for clients”—Mental health or related practitioner

“I have always believed that people with psychosocial disabilities should not be stigmatized and discriminated; and as a result Human Rights should be respected and applied.”—Academic

“The training has reinforced the positive attitude that I have always had towards the mentally ill that no matter the circumstances, they have rights that needs to be respected in all situations.”—Mental health or related practitioner

## Discussion

The current study was the largest study exploring attitude change following the QualityRights training both globally and in Ghana. Encouragingly, most people's attitudes had improved and become more in line with a human-rights based approach in relation to decision making, the use of coercion, the abuse of human rights, community inclusion and perceptions of individuals with mental health conditions and psychosocial and intellectual disabilities. Stereotypes were challenged and there was evidence that attitudes shifted from shifting from viewing individuals as dangerous or dependent to capable and equal. Participants made a clear link between completing the e-training and having improved attitudes.

### Legal capacity and the right to decide

Having autonomy, informed consent and being included in the decision-making process were now all seen as important. There was also a move away from endorsing substitute decision making, with attitudes shifting in favour of providing support to help individuals with lived experience to make decisions rather than imposing care on them. This is consistent with a previous evaluation of the QualityRights face to face training, where attitudinal change relating to the right of those with lived experience to make decisions on their own occurred following training (36). In Ghana, pre-existing attitudes showed that individuals believed that health practitioners and family members were in the best position to make treatment decisions (26). Therefore, attitude change emphasising informed consent, autonomy and supported decisions indicates a shift towards thinking that is in line with the CRPD.

Article 12 of the CRPD, on equal recognition before the law, emphasises the right to make decisions and choices for oneself in all areas of life, with access to support if required, although some aspects of this have been the topic of recent debate (44–46). Furthermore, it prohibits practices which lead to a loss of autonomy in decision making (29, 47). Additionally, articles 14 and 25, emphasise respecting the right of persons with disabilities to liberty and security of the person and the right to consent to medical treatment, while articles 15, 16 and 17 highlight respect for personal integrity and freedom from torture or cruel, inhuman or degrading treatment or punishment, and from violence, exploitation and abuse (47). Informed consent and supported decision making all ensure that decisions are made based on the will and preferences of individuals with lived experience. In line with the quantitative results such attitudinal shifts are a step in the right direction towards a human rights-based approach and a reduction in human rights violations, as there is evidence to suggest that negative attitudes and stereotypes about mental health can result in restrictions to exercise legal capacity and the loss of autonomy, (48). However, despite these apparent positive changes in attitudes there are concerns that without ongoing support there may be some drift back to previous attitudes and practice. This can relate to previous cultural and societal beliefs including stigma as well as potential difficulties making enduring changes in practice where the overall funding for services, staffing, and training remains very low.

### Coercion, violence and abuse

Coercive practices like involuntary treatment and detention were now seen as harmful and unnecessary. Similar attitude shifts towards the use of coercion were reported by Morrissey (36) following QualityRights face to face training, where individuals were more likely to oppose the use of seclusion, restraint and involuntary treatment following QualityRights face to face training.

Attitudes related to the overall treatment of persons with lived experience also improved. While human rights violations and other forms of abuse and violence were previously commonly accepted, they were seen as problematic following e-training. Humane, dignified and respectful treatment for those with lived experience was now preferred and there was the desire to end human rights violations. These attitude shifts are in line with articles 12 and 14 through 17 of the CRPD. Articles 14 which focuses on liberty and security of person, emphasises that there should be no illegal or arbitrary removal of liberties and detainment based on disabilities (3). These articles alongside articles 15, 16 and 17 aim to protect people with lived experience from exploitation, abuse, violence and coercion, forced detention and involuntary treatment (29). Furthermore, article 12 recognises that people have the right to make decisions about their care and treatment, meaning that coercive measures violate this article. When prevailing attitudes support the use of coercion, seeing it as beneficial and necessary, this leads to human rights violations and abuse (13, 49, 50) and within Ghana, the use of coercive measures and abuse has been found within hospital settings and traditional forms of treatment (16–18). The types of attitudinal shifts in this study, towards alignment with the rights of the CRPD, are key to supporting efforts to tackle negative and stigmatising attitudes and practices that influence poor treatment and human rights violations.

### Equality, participation and community inclusion

Shifts in attitudes in relation to equality and community inclusion included perceptions about mental health and people with mental health conditions and their exclusion from society. Feelings about incompetence, threat, risk and recovery had changed, with persons with lived experience being considered as equal members of society, capable of living independently, making decisions, performing tasks and achieving goals and were no longer viewed as dangerous or violent. Additionally, recovery was seen as possible and mental health conditions were no longer seen as self-inflicted. There was increased empathy and understanding and the need for community inclusion through acknowledgement that persons with lived experience should have opportunities to live and work in the community, contribute to society and actively participate in social activities and community life.

Results in relation to equality and community inclusion illustrated a way of thinking that was more human rights oriented and reflective of the equality and social model of disability emphasised within the CRPD. Article 5 of the CRPD promotes equality and non-discrimination while articles 19, 27 and 30 focus on community inclusion, including the right to work, the right to live independently and be included in the community, and building social networks through participation in cultural life, recreation, leisure and sport (29). The move towards a way of thinking which focuses on equality, non-discrimination and community inclusion aligns with the CRPD and can lead to reduced discriminatory practices, increased community participation and inclusion and more positive outlooks on persons with mental health conditions and psychosocial disabilities.

### Attitude change summary

Given the persistence of coercive practices in both formal and informal sectors of Ghanaian mental health care, these findings provide important evidence that structured training can foster more rights-based thinking among diverse stakeholders. Attitude shifts described by participants indicate a more human rights-based way of thinking and a reduction in negative and stigmatising attitudes to mental health and human rights. Moreover, improvements can be seen when compared to the evaluation of pre e-training attitudes which found that attitudes towards the rights of persons with lived experience were not well aligned with a human rights approach to mental health (26). Findings also align with a quantitative analysis of attitude change post e-training where significant attitude changes towards alignment with human rights was found, with scores changing by approximately 40% between baseline and follow-up (43). These improvements in attitudes reflect the e-training's content, it's aims of capacity building and reducing discriminatory and negative attitudes by improving knowledge and transforming mindsets (30) and are consistent with studies examining the impact of the QualityRights face to face training and e-training on successfully improving attitudes towards persons with mental health conditions or psychosocial, intellectual or cognitive disability (35–37). Moreover, the attitudinal improvements found directly reflect the content of the e-training modules and can be linked to its success.

## Limitations

The dataset utilised in the current study provided rich data for analysis. The sample included individuals from many different affiliations and backgrounds. The overall response rate was low compared to the number of people who completed the baseline questionnaires, nevertheless out of the 876 people who completed the follow up questionnaires 716 provided text responses.

Potential researcher bias was also minimised by engaging with other researchers (Author 4, Author 5, Author 6) for reliability checks, to enhance the credibility of the findings. However, there were some methodological problems. Average response length related to attitudinal change was 19 words (130 characters). It is possible that the open questions within the questionnaire as a method of data collection did not allow for an in-depth assessment of attitude change, in the same way that a focus group or interview could have. However, short responses when numerous can reveal important thematic patterns.

Results only represent the attitudes of those who completed the questionnaire, and it is possible that these individuals provided favourable responses or responses that aligned with what was taught in the e-training. Such as desirability cannot be ruled out, but has hopefully been minimised with the online and anonymous nature of the questionnaire.

A small number of participants indicated no shifts in attitudes, primarily because they reported having existing attitudes and beliefs which were positive or human rights-based, or had prior knowledge, and their practice already reflected the recommendations of the e-training.

There is a link between negative attitudes towards mental health and human rights violations in practice (51, 52) and it is therefore possible that these individuals already held human rights-oriented attitudes. Reasons given for lack of change suggest that the QualityRights initiative may be more effective among those without prior human rights-oriented attitudes and behaviour. Furthermore, previous research showed that limited mental health literacy (MHL) can influence negative and stigmatising attitudes (53). In line with this, a lack of human rights-based training among MHPs in Ghana was previously found (18). It may be that MHL acted as a protective factor against the development of negative attitudes, for the participants who stated prior knowledge as a reason for their lack of attitudinal change. While MHL might be a protective factor for negative attitudes, other research has found negative and stigmatising attitudes among health professionals (44, 54), who should have high MHL.

In addition, some participants may not have experienced attitude change and it is possible that for some people attitudinal improvements did occur but there was an unwillingness to admit this, or a lack of awareness recognition that their views had changed. This type of item nonresponse can be seen, as some participants provided open-ended answers to questions related to e-training effects and feedback about the course platform, modules and quality, but did not provide answers to questions related to attitude or practice change. Depending on the time between completion of the e-training and the follow up questionnaire, it is possible that some participants did not yet recognise their attitude change. Perhaps over the course of weeks and months following the training, participants had the time to reappraise their views and come to a new outlook but were not able to recognise these changes at the time they completed the questionnaire. There are also concerns in questionnaire of this nature about social desirability bias, where people knew how to respond the questions because of the QR training, and so some were perhaps unwilling to express any negative views they may have held. This could be more of a concern since people did not give us text comments representing negative attitudes. The authors involved in analysis of the data included two psychiatrists, a psychologist and public health student so it is possible that the personal experiences and work related of these authors could have led to bias on the interpretation of the data, as for example, in their work psychiatrists may be familiar with various human rights issues.

## Future research studies

This study build on our previous quantitative survey (43) which showed that QualityRights training was effective in improving people's attitudes. This paper helps provide a better understanding of the how and why attitudes changed. Further research could look in more depth at the processes of changes for example by in-depth interviews with individuals who completed the e-training which would give participants the opportunity to express their views and perspectives in depth. This would provide a better understanding of barriers and facilitators to attitude change and capture feelings and emotional responses. Follow up work is also needed to ascertain how far the e-training provides sustained attitude change and whether attitudinal improvements reflect better practice. Implementation studies also need to look at how far the e-training can be introduced more widely and mechanisms to work with nations to achieve policy change. Further research could also look in more depth at other human rights approaches beyond the CRPD in relation to good practice in mental health care.

## Conclusion

Taken together, these findings demonstrate widespread attitudinal shifts across legal capacity, coercion, and inclusion, with strong alignment to CRPD principles. The QualityRights e-training is an effective tool in improving attitudes and highlights the success of this initiative in Ghana. It provides a strong evidence base for the utilisation of the QualityRights initiative and e-training to address stigma, discrimination and the use of coercive practices. Embedding QualityRights training into national mental health policy and curricula for health professionals could represent a critical step towards reducing coercion, promoting recovery, and protecting the rights of people with psychosocial and intellectual disabilities across nations. However, it likely that ongoing support and training, and hence additional resources may be needed to do this effectively.

## Data Availability

The raw data supporting the conclusions of this article will be made available by the authors, without undue reservation.
